# A Novel Synthetic Approach to *C*-Glycosyl-d- and l-Alanines

**DOI:** 10.3390/molecules13123171

**Published:** 2008-12-15

**Authors:** Miroslava Martinková, Jozef Gonda, Jana Raschmanová, Alexandra Novodomská, Jozef Kožíšek, Lucia Perasinová

**Affiliations:** 1 Institute of Chemical Sciences, Department of Organic Chemistry, P. J. Šafárik University, Moyzesova 11, SK-040 01 Košice, Slovak Republic; E-mails: jozef.gonda@upjs.sk (J. G.), jrasch@pobox.sk (J. R.), novodomska@pobox.sk (A. N.); 2 Faculty of Chemical and Food Technology, Department of Physical Chemistry, Slovak University of Technology, Radlinského 9, SK-812 37 Bratislava, Slovak Republic; E-mails: jozef.kozisek@stuba.sk (J. K.), lucia.perasinova@stuba.sk (L. P.)

**Keywords:** *C*-Glycosyl amino acids, *C*-Glycosyl alanine, [3,3]-Sigmatropic rearrangements, Microwave irradiation, X-ray diffraction.

## Abstract

*C*-Glycosyl-(*S*)- and (*R*)-alanines **12a** and **12b** were synthesized from the known β-*C*-glycoside **1**. The nitrogen function was introduced by aza-Claisen rearrangement of the allylic thiocyanate **7**, derived from the corresponding alcohol **6**. The absolute configuration of the newly created chiral carbon center (C-3) was assigned by X-ray diffraction analysis of the intermediate 3(*S*)-isothiocyanato-d-*glycero*-d-*galacto*-decose **8a**.

## Introduction

Glycoconjugates [1] have a significant pharmaceutical potential and intensive research on understanding the functions of these structures in biological events has become a major target for many scientific groups in the recent years.

This increasing interest has been recently turned to modified glycosyl amino acids such as *C*-glycosyl α-amino acids or fused sugar amino acids [2,3,4,5], in which carbohydrate and amino acid are linked directly to the anomeric centre of the sugar either via a carbon-carbon bond or an entire α-amino acid (glycinyl moiety) [2]. They represent a significant class of building blocks for the construction of *C*-glycosylated peptides [3,6]. The incorporation of unnatural *C*-glycosyl amino acids in glycopeptide mimetics may serve for preparing analogues with enhanced resistance to enzymatic hydrolysis but also in the development of glycopeptide-based drugs with interesting pharmacological properties [3,4,5]. For the construction of the *C*-glycosyl amino acids, several synthetic approaches have been developed [2,5,6,7,8,9,10].

## Results and Discussion

**Scheme 1:**
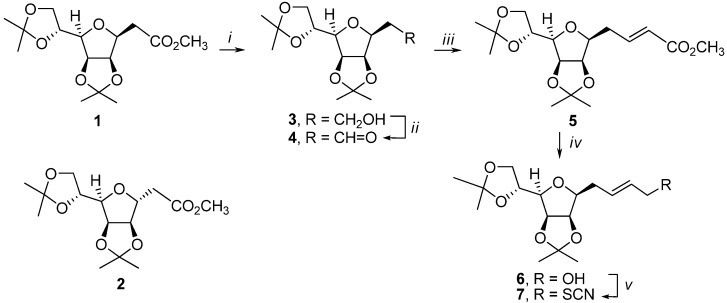
Synthesis of protected 1-thiocyanato-d-*glycero*-d-*galacto*-dec-2(*E*)-enitol **7**.

We report here a synthetic strategy for the preparation of diastereomerically pure *C*-glycosyl-alanines **12a**, **12b**, starting from the know β-*C*-glycoside **1** [11] and based on the aza-Claisen rearrangement of allylic thiocyanates previously developed in our laboratory [12,13]. The starting β-*C*-glycoside **1** was synthesized together with its α-anomer **2** via a Wittig-intramolecular Michael-type ring closure sequence from the known 2,3:5,6-di-*O*-isopropylidene-α-d-mannofuranose and a stabilized ylide (Ph_3_P=CHCO_2_CH_3, _acetonitrile, reflux). Subsequent reduction of **1** with lithium aluminum hydride in dry diethyl ether gave alcohol **3** (88%, Scheme 1). The 2,5-anhydroalditol **3** was subsequently oxidized with *o*-iodoxybenzoic acid [14] (IBX) in acetonitrile to yield the corresponding aldehyde **4** in 93% yield. The aldehyde **4** was then treated with the stabilized ylide Ph_3_P=CHCO_2_CH_3_ to afford (*E*)-α,β-unsaturated ester **5** in 87% yield (Scheme 1). 

*Reagents and conditions*: (*i*) LiAlH_4_, Et_2_O; (*ii*) IBX, CH_3_CN; (*iii*) Ph_3_P=CHCO_2_CH_3_, CH_2_Cl_2_; (*iv*) DIBAH, CH_2_Cl_2_; (*v*) MsCl, Et_3_N/KSCN, CH_3_CN.

**Scheme 2:**
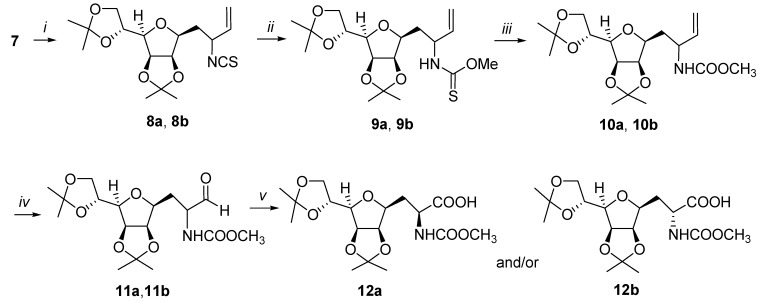
Synthesis of *C*-glycosyl-(*S*)- and (*R*)-alanines.

Its structure was determined by ^1^H- and ^13^C-NMR spectroscopy (for data see Experimental part). The observed coupling constant in **5** (*J*_3,2_ = 15.7 Hz) accounted for a *trans*-configuration of the double bond. The ester **5** was subjected to reduction with diisobutylaluminum hydride in CH_2_Cl_2_ to give the allylic alcohol **6** (75%). The required thiocyanate **7** was easily prepared in 76% overall yield by a two-step process of mesylation of alcohol **6** followed by displacement using KSCN in acetonitrile (Scheme 1). The thermal aza-Claisen rearrangement of thiocyanate **7**, which was carried out at 90 ^o^C in dry *n*-heptane under a nitrogen atmosphere for 6 h, afforded a mixture of diastereomeric isothiocyanates **8a** and **8b **(Scheme 2), with high yield (83%) but without selectivity (**8a**:**8b** ≈ 1:1, as determined by ^1^H- NMR). The microwave (MW) induced rearrangement of thiocyanate **7** realized under the same conditions (90 ^o^C, *n*-heptane, Scheme 2) gave a 1:1 mixture of **8a **and **8b** in 86% yield, within 2 h. The reaction was performed in closed vessel in a focused microwave reactor (CEM Discover, see Experimental part). 

**Figure 1:**
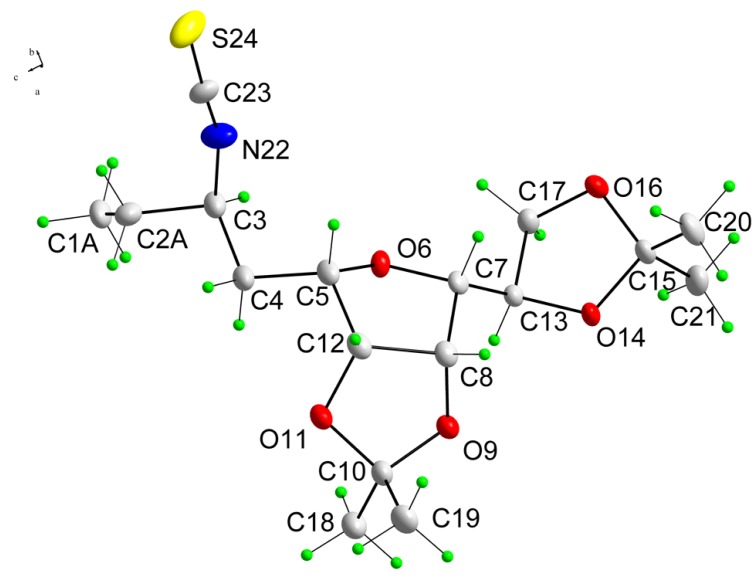
A molecular structure of **8a**, showing crystallographic numbering.

We have observed that the use of microwave irradiation remarkably accelerated rearrangement of **7→8a**, **8b **with reduction to one-third of the reaction time, in comparison with the conventional thermal conditions, but it had practically no influence on the selectivity of the rearrangement. 

Fortunately, these diastereoisomers were easily separated by chromatography and compound **8a** was isolated in crystalline state. In order to determine the absolute configuration of compound **8a**, we tried to recrystallize **8a** to obtain single crystals for X-ray diffraction analysis. The isothiocyanate **8a** crystallized well from a mixture of ether and hexane, forming colorless prisms suitable for X-ray measurements. The crystallographic structure of compound **8a**, shown in Figure 1, confirmed that the newly introduced stereocentre at C-3 in **8a** possesses *S* configuration. Consequently, the isothiocyanate **8b** must be the 3*R*-epimer.

*Reagents and conditions*: (*i*) a) *n*-heptane, 90 °C b) *n*-heptane, 90 °C, microwaves; (*ii*) CH_3_ONa, CH_3_OH; (*iii*) MNO, CH_3_CN; (*iv*) O_3_, -78 °C, Ph_3_P; (*v*) NaClO_2_, CH_3_CN/*t*-BuOH/2-methylbut-2-ene = 4:4:1.

Our approach to the build-up of *C*-glycosyl-(*S*)- and (*R*)-alanines **12a** and **12b** was based on four subsequent steps which were conducted with pure diastereoisomers **8a** and **8b**. In the first step, the reaction of **8a** and **8b** with CH_3_ONa in dry methanol at room temperature gave a nearly quantitative yield of thiourethanes **9a** and **9b**, which were used immediately in the next step without purification to avoid problems connected with their possible instability. The treatment of **9a** and **9b** with mesitonitrile oxide (MNO) [15] in acetonitrile afforded in 85% and 92% yields, respectively, carbamates **10a** and **10b **(Scheme 2), whose structure was confirmed by ^1^H- and ^13^C-NMR spectroscopy (for data see experimental part). Ozonolysis of **10a** and **10b** at -78 ^o^C in methanol afforded the corresponding aldehydes **11a** and **11b**. After a short pad filtration on silica gel (to remove arising triphenylphosphine oxide), these products were used immediately in the next step due to instability of α-amino aldehydes. The structure of **11a** and **11b** was determined by ^1^H-NMR; the observed chemical shift of aldehyde proton in **11a** δ = 9.64 ppm and in **11b** δ = 9.52 ppm. The aldehydes **11a** and **11b** were selectively oxidized to protected *C*-glycosyl-(*S*)- and (*R*)-alanines **12a** and **12b **(Scheme 2) by treatment with sodium chlorite (NaClO_2_) in CH_3_CN/*tert*-butyl alcohol/2-methyl-2-butene at 0 ^o^C in 74% and 73% yields, respectively after flash chromatography.

## Conclusions

In summary, the novel synthetic approach to the chiral non-racemic *C*-glycosylated alanines **12a** and **12b** has been developed. The obtained compounds **12a** and **12b** differ in the stereochemistry of the newly formed chiral carbon atom (C-2), one having the l-configuration (**12a**) and the other the d-configuration (**12b**). These novel amino acids **12a** and **12b** can be useful in modifying the properties of some glycopeptides by virtue of the presence of a stable anomeric C-C bond instead of the C-O or C-N bond and an additional amino group at C-2.

## Experimental

### General

All commercially available reagents were used without purification and solvents were dried according to standard procedures. Product purification was carried out using flash chromatography on silica gel (Merck silica gel 60 (0.040-0.063 mm). TLC was run on Merck silica gel 60 F_254_ analytical plates; detection was carried out with either UV, iodine and spraying with a solution of KMnO_4_, with subsequent heating. The melting points were determined on the Kofler block, and are uncorrected. Optical rotations were measured in chloroform, using a P3002 Krüss polarimeter and reported as follows: [α]_d_^25^ (*c* in g/100 mL, solvent). NMR spectra were recorded at room temperature on a FT NMR spectrometer Varian Mercury Plus 400 (^1^H at 400.13 MHz and ^13^C at 100.6 MHz) using CDCl_3_ as the solvent and TMS as internal reference. For ^1^H δ are given in parts per million relative to TMS (0 ppm), for ^13^C relative to CDCl_3_ (77 ppm). ^13^C-NMR multiplicities were determined by a DEPT pulse sequence. IR spectra were recorded on a Perkin-Elmer 599 IR spectrometer in CHCl_3._ All reactions were performed under nitrogen atmosphere when anhydrous solvents were used. Microwave experiments were conducted using a focused microwave system (CEM Discover). All experiments were performed in glass vessels (10 mL) sealed with a septum. At the end of reaction, the vessels and contents were cooled rapidly using a stream of compressed air.

*Methyl 3,6-anhydro-2-deoxy-4,5:7,8-di-O-isopropylidene-d-glycero-d-galacto-octanoate* (**1**) and *Methyl 3,6-anhydro-2-deoxy-4,5:7,8-di-O-isopropylidene-d-glycero-d-talo-octanoate* (**2**). **1**: ^1^H-NMR: δ 1.33 (3H, s, CH_3_), 1.37 (3H, s, CH_3_), 1.44 (3H, s, CH_3_), 1.46 (3H, s, CH_3_), 2.73 (1H, dd, *J*_2,2_=16.7 Hz, *J*_3,2_=6.4 Hz, H_2_), 2.81 (1H, dd, *J*_2,2_=16.7 Hz, *J*_3,2_=7.3 Hz, H_2_), 3.52 (1H, m, H_6_), 3.70 (3H, s, OCH_3_), 3.94 (1H, m, H_3_), 4.04 (1H, dd, *J*_8,8_=8.7 Hz, *J*_8,7_=4.7 Hz, H_8_), 4.07 (1H, dd, *J*_8,8_=8.7 Hz, *J*_8,7_=6.1 Hz, H_8_), 4.38 (1H, ddd, *J*_7,6_=7.5 Hz, *J*_8,7_=6.1 Hz, *J*_8,7_=4.7 Hz, H_7_), 4.76 (2H, m, H_4_, H_5_); ^13^C- NMR: δ 24.6, 25.2, 25.7, 26.9, 33.3, 51.8, 66.9, 73.1, 77.7, 80.7, 81.0, 81.6, 109.1, 112.6, 171.4. The procedure and [α]_D_ were consistent with those reported [11]. **2**: ^1^H-NMR: δ 1.34 (3H, s, CH_3_), 1.37 (3H, s, CH_3_), 1.45 (3H, s, CH_3_), 1.51 (3H, s, CH_3_), 2.47 (1H, dd, *J*_2,2_=15.2 Hz, *J*_3,2_=7.1 Hz, H_2_), 2.54 (1H, dd, *J*_2,2_=15.2 Hz, *J*_3,2_=7.7 Hz, H_2_), 3.71 (3H, s, OCH_3_), 3.79 (1H, dd, *J*_7,6_=7.7 Hz, *J*_6,5_=3.7 Hz, H_6_), 4.00 (1H, dd, *J*_8,8_=8.7 Hz, *J*_8,7_=4.4 Hz, H_8_), 4.08 (1H, dd, *J*_8,8_=8.7 Hz, *J*_8,7_=6.3 Hz, H_8_), 4.39 (1H, ddd, *J*_7,6_=7.7 Hz, *J*_8,7_=6.3 Hz, *J*_8,7_=4.4, H_7_), 4.49 (1H, dd, *J*_3,2_=7.7 Hz, *J*_3,2_=7.1 Hz, H_3_), 4.64 (1H, d, *J*_5,4_=6.0 Hz, H_4_), 4.81 (1H, dd, *J*_5,4_=6.0 Hz, *J*_6,5_=3.7 Hz, H_5_); ^13^C-NMR: δ 24.7, 25.2, 26.1, 27.0, 36.2, 51.9, 66.9, 73.3, 80.8, 80.9, 80.9, 84.9, 109.2, 112.9, 170.6. The procedure, m.p. and [α]_d_ were consistent with those reported [11]. 

*3,6-Anhydro-2-deoxy-4,5:7,8-di-O-isopropylidene-d-glycero-d-galacto-octitol* (**3**): LiAlH_4_ (0.87 g, 23.0 mmol) was added at 0 ^o^C to a solution of ester **1** (3.83 g, 12.1 mmol) in dry Et_2_O (70 mL). The reaction mixture was stirred at 0 ^o^C for 15 min and then for 45 min at room temperature. The reaction was quenched by careful addition of water (3 mL) and the precipitate was removed by filtration. The filtrate was dried (Na_2_SO_4_) and concentrated under reduced pressure. The chromatography of the residue on silica gel (hexane-ethyl acetate, 2:1) afforded 3.03 g (88%) of alcohol **3 **as a colorless oil; [α]_d_^25^ = -23 (*c* 0.49, CHCl_3_); ^1^H-NMR: δ 1.34 (3H, s, CH_3_), 1.38 (3H, s, CH_3_), 1.44 (3H, s, CH_3_), 1.48 (3H, s, CH_3_), 1.93 (1H, m, H_2_), 2.05 (1H, m, H_2_), 3.54 (1H, dd, *J*_7,6_=7.2 Hz, *J*_6,5_=3.7 Hz, H_6_), 3.70 (1H, ddd, *J*_3,2_=8.4 Hz, *J*_3,2_=5.1 Hz, *J*_4,3_=3.7 Hz, H_3_), 3.80 (2H, m, H_1_), 4.05 (1H, dd, *J*_8,8_=8.7 Hz, *J*_8,7_=4.8 Hz, H_8_), 4.09 (1H, dd, *J*_8,8_=8.7 Hz, *J*_8,7_=6.1 Hz, H_8_), 4.40 (1H, m, H_7_), 4.66 (1H, dd, *J*_5,4_=6.1 Hz, *J*_4,3_=3.7 Hz, H_4_), 4.76 (1H, dd, *J*_5,4_=6.1 Hz, *J*_6,5_=3.7 Hz, H_5_); ^13^C-NMR: δ 24.6, 25.3, 25.7, 26.9, 31.1, 60.4, 66.8, 73.1, 80.5, 80.6, 81.7, 81.7, 109.0, 112.4; Anal. Calcd for C_14_H_24_O_6_ (288.34): C 58.32, H 8.39; found C 58.54, H 8.66.

*3,6-Anhydro-2-deoxy-4,5:7,8-di-O-isopropylidene-d-glycero-d-galacto-octose* (**4**): To a solution of alcohol **3 **(3.0 g, 10.5 mmol) in CH_3_CN (55 mL) was added IBX (4.41 g, 15.7 mmol). The resulting suspension was heated under reflux for 40 min. Then the reaction was cooled to room temperature and filtered through a medium glass frit. The filter cake was washed with further portions of acetonitrile (2 x 15 mL). The combined filtrates were concentrated under reduced pressure. The chromatography of the residue on silica gel (hexane-ethyl acetate, 3:1) afforded 2.73 g (93%) of aldehyde **4 **as a colorless oil; [α]_d_^25^ = -12 (*c* 0.68, CHCl_3_); ^1^H-NMR: δ 1.32 (3H, s, CH_3_), 1.38 (3H, s, CH_3_), 1.45 (3H, s, CH_3_), 1.46 (3H, s, CH_3_), 2.87-2.89 (2H, m, H_2_), 3.55 (1H, dd, *J*_7,6_=7.4 Hz, *J*_6,5_=3.3 Hz, H_6_), 3.99 (1H, ddd, *J*_3,2_=6.4 Hz, *J*_3,2_=6.4 Hz, *J*_4,3_=3.3 Hz, H_3_), 4.03 (1H, dd, *J*_8,8_=8.7 Hz, *J*_8,7_=4.7 Hz, H_8_), 4.08 (1H, dd, *J*_8,8_=8.7 Hz, *J*_8,7_=6.2 Hz, H_8_), 4.39 (1H, ddd, *J*_7,6_=7.4 Hz, *J*_8,7_=6.2 Hz, *J*_8,7_=4.7 Hz, H_7_), 4.76 (1H, dd, *J*_5,4_=6.1 Hz, *J*_4,3_=3.3 Hz, H_4_), 4.79 (1H, dd, *J*_5,4_=6.1 Hz, *J*_6,5_=3.3 Hz, H_5_), 9.81 (1H, t, *J*=1.3 Hz, CHO); ^13^C-NMR: δ 24.5, 25.2, 25.6, 26.9, 42.8, 66.8, 73.0, 76.6, 80.6, 81.0, 81.6, 109.1, 112.6, 199.9; Anal. Calcd for C_14_H_22_O_6_ (286.33): C 58.73, H 7.74; found C 58.49, H 7.51.

*Methyl 5,8-anhydro-2,3,4-trideoxy-6,7:9,10-di-O-isopropylidene-d-glycero-d-galacto-dec-2(E)-enoate* (**5**): [(Methoxycarbonyl)methylidene]triphenylphosphorane (3.82 g, 11.4 mmol) was added to a solution of aldehyde **4** (2.73 g, 9.5 mmol) in dry CH_2_Cl_2_ (25 mL). The reaction mixture was stirred for 1.5 h at room temperature. The solvent was removed under reduced pressure and the residue was purified by chromatography on silica gel (hexane-ethyl acetate, 5:1) to afford 2.79 g (87%) of (*E*)-**5** as a colorless oil; [α]_d_^25 ^= -12 (*c* 0.34, CHCl_3_); ^1^H-NMR: δ 1.33 (3H, s, CH_3_), 1.38 (3H, s, CH_3_), 1.44 (3H, s, CH_3_), 1.48 (3H, s, CH_3_), 2.61 (2H, m, H_4_), 3.50 (1H, dd, *J*_9,8_=7.5 Hz, *J*_8,7_=3.6 Hz, H_8_), 3.59 (1H, ddd, *J*_5,4_=6.8 Hz, *J*_5,4_=6.8 Hz, *J*_6,5_=3.6 Hz, H_5_), 3.73 (3H, s, OCH_3_), 4.05 (1H, dd, *J*_10,10_=8.7 Hz, *J*_10,9_=4.8 Hz, H_10_), 4.09 (1H, dd, *J*_10,10_=8.7 Hz, *J*_10,9_=6.1 Hz, H_10_), 4.39 (1H, ddd, *J*_9,8_=7.5 Hz, *J*_10,9_=6.1 Hz, *J*_10,9_=4.8 Hz, H_9_), 4.63 (1H, dd, *J*_7,6_=6.1 Hz, *J*_6,5_=3.6 Hz, H_6_), 4.76 (1H, dd, *J*_7,6_=6.1 Hz, *J*_8,7_=3.6 Hz, H_7_), 5.94 (1H, ddd, *J*_3,2_=15.7 Hz, *J*_4,2_=1.5 Hz, *J*_4,2_=1.5 Hz, H_2_), 6.99 (1H, ddd, *J*_3,2_=15.7 Hz, *J*_4,3_=7.1 Hz, *J*_4,3_=7.1 Hz, H_3_); ^13^C-NMR: δ 24.6, 25.3, 25.7, 26.9, 31.4, 51.5, 66.9, 73.1, 80.3, 80.7, 81.1, 81.7, 109.1, 112.6, 123.0, 144.9, 166.8; Anal. Calcd for C_17_H_26_O_7_ (342.39): C 59.64, H 7.65; found C 59.73, H 7.79.

*5,8-Anhydro-2,3,4-trideoxy-6,7:9,10-di-O-isopropylidene-d-glycero-d-galacto-dec-2(E)-enitol* (**6**): To a solution of ester **5** (2.79 g, 8.15 mmol) in dry CH_2_Cl_2 _(37 mL) diisobutylaluminum hydride (24.6 mL of 1.2 M toluene solution) was added dropwise at -15 °C. The resulting mixture was stirred for 45 min at the same temperature and then quenched with methanol (6.2 mL). The mixture was allowed to warm to room temperature and poured into 30% aqueous K/Na tartrate (123 mL). After stirring for 30 min, the product was extracted with CH_2_Cl_2_ (3 x 37 mL). The combined organic layers were dried (Na_2_SO_4_) and the solvent evaporated under reduced pressure. Chromatography of the residue on silica gel (hexane-ethyl acetate, 1:1) afforded 2.34 g (91%) of allylic alcohol **6** as a colorless oil; [α]_d_^25^ = +22 (*c* 0.28, CHCl_3_); ^1^H-NMR: δ 1.34 (3H, s, CH_3_), 1.38 (3H, s, CH_3_), 1.45 (3H, s, CH_3_), 1.48 (3H, s, CH_3_), 2.47 (2H, m, H_4_), 3.48 (1H, m, H_8_), 3.52 (1H, m, H_5_), 4.05 (1H, dd, *J*_10,10_=8.7 Hz, *J*_10,9_=4.8 Hz, H_10_), 4.08 (1H, dd, *J*_10,10_=8.7 Hz, *J*_10,9_=6.0 Hz, H_10_), 4.11 (2H, m, H_1_), 4.40 (1H, ddd, *J*_9,8_=7.4 Hz, *J*_10,9_=6.0 Hz, *J*_10,9_=4.8 Hz, H_9_), 4.62 (1H, dd, *J*_7,6_=6.1 Hz, *J*_6,5_=3.6 Hz, H_6_), 4.74 (1H, dd, *J*_7,6_=6.1 Hz, *J*_8,7_=3.7 Hz, H_7_), 5.76 (2H, m, H_2_, H_3_); ^13^C-NMR: δ 24.7, 25.3, 25.8, 26.9, 31.2, 63.6, 66.9, 73.2, 80.7, 81.1, 81.5, 81.6, 109.0, 112.4, 128.2, 131.5; Anal. Calcd for C_16_H_26_O_6_ (314.38): C 61.13, H 8.34; found C 61.32, H 8.50. 

*5,8-Anhydro-1,2,3,4-tetradeoxy-6,7:9,10-di-O-isopropylidene-1-thiocyanato-d-glycero-d-galacto-dec-2(E)-enitol* (**7**): To a solution of alcohol **6** (2.34 g, 7.44 mmol) in dry dichloromethane (26 mL) were added triethylamine (1.55 mL, 11.17 mmol) and CH_3_SO_2_Cl (0.69 mL, 8.93 mmol) at 0 ^o^C. The mixture was stirred at 0 ^o^C for 15 min and then further 45 min at room temperature. The solvent was evaporated under reduced pressure. The residue was diluted with diethyl ether (40 mL) and the solid was removed by filtration. The solvent was evaporated to afford the crude mesylate which was used in the subsequent reaction directly without further purification. To the crude mesylate dissolved in CH_3_CN (26 mL), KSCN (1.09 g, 11.17 mmol) was added. After stirring at room temperature for 1 h, the solvent was evaporated. The residue was diluted with diethyl ether (40 mL) and the solid was removed by filtration. Evaporation of the solvent and chromatography of the residue (hexane-ethyl acetate, 5:1) afforded 2.0 g (76%) of thiocyanate **7** as white crystals; m.p. 81–82 °C; [α]_d_^25^ = +23 (*c* 0.28, CHCl_3_); ^1^H-NMR: δ 1.34 (3H, s, CH_3_), 1.38 (3H, s, CH_3_), 1.44 (3H, s, CH_3_), 1.48 (3H, s, CH_3_), 2.50-2.54 (2H, m, H_4_), 3.49 (1H, dd, *J*_9,8_=7.5 Hz, *J*_8,7_=3.7 Hz, H_8_), 3.52-3.55 (3H, m, 2 x H_1_, H_5_), 4.05 (1H, dd, *J*_10,10_=8.7 Hz, *J*_10,9_=4.8 Hz, H_10_), 4.09 (1H, dd, *J*_10,10_=8.7 Hz, *J*_10,9_=6.1 Hz, H_10_), 4.39 (1H, ddd, *J*_9,8_=7.5 Hz, *J*_10,9_= 6.1 Hz, *J*_10,9_=4.8 Hz, H_9_), 4.67 (1H, dd, *J*_7,6_=6.1 Hz, *J*_6,5_=3.6 Hz, H_6_), 4.74 (1H, dd, *J*_7,6_=6.1 Hz, *J*_8,7_=3.7 Hz, H_7_), 5.70 (1H, m, H_2_), 5.87 (1H, m, H_3_); ^13^C-NMR: δ 24.6, 25.3, 25.8, 26.9, 31.4, 36.3, 66.9, 73.1, 80.7 81.0, 81.2, 81.6, 109.0, 111.9, 112.4, 124.9, 134.0; Anal. Calcd for C_17_H_25_NO_5_S (355.46): C 57.44, H 7.09, N 3.94; found C 57.61, H 7.28, N 4.04.

*5,8-Anhydro-1,2,3,4-tetradeoxy-6,7:9,10-di-O-isopropylidene-3(S)-isothiocyanato-d-glycero-d-galacto-dec-1-enitol* (**8a**) and *5,8-Anhydro-1,2,3,4-tetradeoxy-6,7:9,10-di-O-isopropylidene-3(R)-isothiocyanato-d-glycero-d-galacto-dec-1-enitol* (**8b**): *Conventional method for the preparation of* **8a**, **8b**: A solution of thiocyanate **7** (1.80 g, 5.06 mmol) in dry *n*-heptane (30 mL) was heated at 90 ^o^C for 6 h under nitrogen atmosphere. The solvent was evaporated under reduced pressure. The chromatography of the residue on silica gel (hexane-ethyl acetate, 9:1) afforded isothiocyanates **8a **(0.75 g, 42%) and **8b** (0.73 g, 41%). 

*Microwave-assisted synthesis of* **8a**, **8b**: The (*E*)-thiocyanate **7** (20 mg, 0.056 mmol) was weighed in a 10 ml glass pressure microwave tube equipped with a magnetic stirrer bar. Dry *n*-heptane (0.4 mL) was added, the tube was closed with a silicon septum and the reaction mixture was subjected to microwave irradiation for 2 h (power: 150 W, temperature: 90 ^o^C, pressure: 12 bar). The reaction mixture was allowed to cool to room temperature and transferred into a round bottom flask. The solvent was evaporated under reduced pressure. The chromatography of the residue on silica gel (hexane-ethyl acetate, 9:1) gave 0.16 mg (86%) of isothiocyanates **8a**, **8b**. *Compound* **8a**: white crystals; m.p. 54-56 ^o^C; [α]_d_^25^ = -19 (*c* 0.27, CHCl_3_); υ_max_ (liquid film) 2033 (NCS) cm^-1^; ^1^H-NMR: δ 1.33 (3H, s, CH_3_), 1.38 (3H, s, CH_3_), 1.45 (3H, s, CH_3_), 1.46 (3H, s, CH_3_), 1.93 (1H, m, H_4_), 2.07 (1H, ddd, *J*_4,4_=14.3 Hz, *J*_5,4_=9.3 Hz, *J*_4,3_=3.7 Hz, H_4_), 3.54 (1H, dd, *J*_9,8_=7.6 Hz, *J*_8,7_=3.7 Hz, H_8_), 3.71 (1H, ddd, *J*_5,4_=9.3 Hz, *J*_6,5_=3.7 Hz, *J*_5,4_=3.5 Hz, H_5_), 4.06 (1H, dd, *J*_10,10_=8.7 Hz, *J*_10,9_=4.8 Hz, H_10_), 4.09 (1H, dd, *J*_10,10_=8.7 Hz, *J*_10,9_=6.0 Hz, H_10_), 4.39 (1H, ddd, *J*_9,8_=7.6 Hz, *J*_10,9_=6.0 Hz, *J*_10,9_=4.8 Hz, H_9_), 4.45 (1H, m, H_3_), 4.64 (1H, dd, *J*_7,6_=6.1 Hz, *J*_6,5_=3.7 Hz, H_6_), 4.78 (1H, dd, *J*_7,6_=6.1 Hz, *J*_8,7_=3.7 Hz, H_7_), 5.24 (1H, dd, *J*_2,1*cis*_=10.2 Hz, *J*_3,1*cis*_=1.4 Hz, H_1*cis*_), 5.39 (1H, dd, *J*_2,1*trans*_=16.9 Hz, *J*_3,1*trans*_=1.6 Hz, H_1*trans*_), 5.83 (1H, ddd, *J*_2,1*trans*_=16.9 Hz, *J*_2,1*cis*_=10.2 Hz, *J*_3,2_=5.4 Hz, H_2_); ^13^C-NMR: δ 24.5, 25.3, 25.7, 26.9, 35.3, 57.5, 66.9, 73.0, 78.1, 80.8, 81.5, 81.7, 109.2, 112.5, 116.5, 132.9, 135.2; Anal. Calcd for C_17_H_25_NO_5_S (355.46): C 57.44, H 7.09, N 3.94; found C 57.25, H 7.20, N 4.11. *Compound* **8b**: a colorless oil; [α]_d_^25^ = -10 (*c* 0.41, CHCl_3_); υ_max_ (liquid film) 2020 (NCS) cm^-1^; ^1^H-NMR: δ 1.33 (3H, s, CH_3_), 1.38 (3H, s, CH_3_), 1.44 (3H, s, CH_3_), 1.47 (3H, s, CH_3_), 2.03 (1H, m, H_4_), 2.16 (1H, m, H_4_), 3.49 (1H, dd, *J*_9,8_=7.6 Hz, *J*_8,7_=3.6 Hz, H_8_), 3.61 (1H, ddd, *J*_5,4_=7.4 Hz, *J*_5,4_=6.1 Hz, *J*_6,5_=3.7 Hz, H_5_), 4.05 (1H, dd, *J*_10,10_=8.8 Hz, *J*_10,9_=4.6 Hz, H_10_), 4.09 (1H, dd, *J*_10,10_=8.8 Hz, *J*_10,9_=6.2 Hz, H_10_), 4.36–4.41 (2H, m, H_3_, H_9_), 4.64 (1H, dd, *J*_7,6_=6.1 Hz, *J*_6,5_=3.7 Hz, H_6_), 4.77 (1H, dd, *J*_7,6_=6.1 Hz, *J*_8,7_=3.6 Hz, H_7_), 5.25 (1H, ddd, *J*_2,1´*cis*_=10.2 Hz, *J*_3,1*cis*_=1.1 Hz, *J*_1*cis*,1*trans*_=0.5 Hz, H_1*cis*_), 5.36 (1H, ddd, *J*_2,1*trans*_=16.8 Hz, *J*_3,1*trans*_=1.3 Hz, *J*_1*cis*,1*trans*_=0.5 Hz, H_1*trans*_), 5.81 (1H, ddd, *J*_2,1*trans*_=16.8 Hz, *J*_2,1*cis*_=10.2 Hz, *J*_3,2_=6.0 Hz, H_2_); ^13^C-NMR: δ 24.6, 25.2, 25.7, 27.0, 34.8, 57.3, 66.9, 73.0, 78.4, 80.7, 81.0, 81.7, 109.1, 112.6, 117.2, 132.7, 134.8; Anal. Calcd for C_17_H_25_NO_5_S (355.46): C 57.44, H 7.09, N 3.94; found C 57.62, H 6.94, N 3.88.

*5,8-Anhydro-1,2,3,4-tetradeoxy-6,7:9,10-di-O-isopropylidene-3(S)-(methoxycarbonylamino)-d-glycero-d-galacto-dec-1-enitol* (**10a**): To a solution of isothiocyanate **8a** (0.54 g, 1.52 mmol) in dry methanol (15 mL) was added sodium methoxide (90 mg, 1.67 mmol). The reaction mixture was stirred for 3 h at room temperature under nitrogen atmosphere. The solvent was evaporated and the residue was partitioned between CH_2_Cl_2_ (25 mL) and water (7 mL). The organic layer was dried (Na_2_SO_4_) and the solvent was evaporated under reduced pressure to provide the crude thiourethane **9a** which was used in the subsequent reaction directly without further purification. To a solution of **9a** (436 mg, 1.12 mmol) in dry acetonitrile (10.8 mL) was added mesitonitrile oxide (218 mg, 1.35 mmol). The mixture was stirred at room temperature for 2 h under nitrogen atmosphere. The solvent was evaporated under reduced pressure. The chromatography of the residue (hexane-ethyl acetate, 3:1) gave 0.35 g (85%) of **10a** as a colorless oil; ^1^H-NMR: δ 1.33 (3H, s, CH_3_), 1.38 (3H, s, CH_3_), 1.45 (3H, s, CH_3_), 1.47 (3H, s, CH_3_), 1.88 (1H, m, H_4_), 2.04 (1H, m, H_4_), 3.51 (1H, dd, *J*_9,8_=6.7 Hz, *J*_8,7_=3.7 Hz, H_8_), 3.63 (1H, m, H_5_), 3.66 (3H, s, CH_3_O), 4.05 (1H, dd, *J*_10,10_=8.7 Hz, *J*_10,9_=4.8 Hz, H_10_), 4.10 (1H, dd, *J*_10,10_=8.7 Hz, *J*_10,9_=6.2 Hz, H_10_), 4.36-4.41 (2H, m, H_3_, H_9_), 4.59 (1H, dd, *J*_7,6_=6.1 Hz, *J*_6,5_=3.7 Hz, H_6_), 4.72 (1H, dd, *J*_7,6_=6.1 Hz, *J*_8,7_=3.7 Hz, H_7_), 5.13 (1H, d, *J*_2,1*cis*_=10.4 Hz, H_1*cis*_), 5.20 (1H, d, *J*_2,1*trans*_=17.1 Hz, H_1*trans*_), 5.38 (1H, d, *J*_3,NH_=6.4 Hz, NH), 5.80 (1H, ddd, *J*_2,1*trans*_=17.1 Hz, *J*_2,1*cis*_=10.4 Hz, *J*_3,2_=5.1 Hz, H_2_); ^13^C- NMR: δ 24.5, 25.3, 25.7, 26.9, 32.6, 51.0, 52.0, 66.8, 73.1, 79.1, 80.5, 81.7, 81.8, 109.1, 112.5, 114.7, 138.2, 156.4; Anal. Calcd for C_18_H_29_NO_7_ (371.43): C 58.21, H 7.87, N 3.77; found C 58.46, H 7.61, N 3.92.

*5,8-Anhydro-1,2,3,4-tetradeoxy-6,7:9,10-di-O-isopropylidene-3(R)-(methoxycarbonylamino)-d-glycero-d-galacto-dec-1-enitol* (**10b**): To a solution of isothiocyanate **8b** (416 mg, 1.17 mmol) in dry methanol (11.6 mL) was added sodium methoxide (69.5 mg, 1.29 mmol). The reaction mixture was stirred for 4 h at room temperature under nitrogen atmosphere. The solvent was evaporated and the residue was partitioned between CH_2_Cl_2_ (20 mL) and water (6 mL). The organic layer was dried (Na_2_SO_4_). The solvent was evaporated under reduced pressure to give the crude thiourethane **9b **which was used in the subsequent reaction directly without further purification. To a solution of **9b** (288 mg, 0.74 mmol) in dry acetonitrile (7 mL) was added mesitonitrile oxide (144 mg, 0.89 mmol). The mixture was stirred at room temperature for 2 h under nitrogen atmosphere. The solvent was evaporated under reduced pressure and the chromatography of the residue (hexane-ethyl acetate, 3:1) afforded 0.25 g (92%) of **10b** as a colorless oil; ^1^H-NMR: δ 1.33 (3H, s, CH_3_), 1.37 (3H, s, CH_3_), 1.44 (3H, s, CH_3_), 1.47 (3H, s, CH_3_), 1.84 (1H, ddd, *J*_4,4_=14.2 Hz, *J*_4,3_=9.5 Hz, *J*_5,4_=6.5 Hz, H_4_), 2.00 (1H, m, H_4_), 3.47 (1H, dd, *J*_9,8_=7.5 Hz, *J*_8,7_=3.6 Hz, H_8_), 3.59 (1H, ddd, *J*_5,4_=6.5 Hz, *J*_5,4_=6.5 Hz, *J*_6,5_=3.6, H_5_), 3.67 (3H, s, CH_3_O), 4.03 (1H, dd, *J*_10,10_=8.7 Hz, *J*_10,9_=4.6 Hz, H_10_), 4.08 (1H, dd, *J*_10,10_=8.7 Hz, *J*_10,9_=6.2 Hz, H_10_), 4.29 (1H, m, H_3_), 4.38 (1H, ddd, *J*_9,8_=7.5 Hz, *J*_10,9_=6.2 Hz, *J*_10,9_=4.6 Hz, H_9_), 4.67 (1H, dd, *J*_7,6_=6.1 Hz, *J*_6,5_=3.6 Hz, H_6_), 4.73 (1H, dd, *J*_7,6_=6.1 Hz, *J*_8,7_=3.6 Hz, H_7_), 4.93 (1H, m, NH), 5.12 (1H, dd, *J*_2,1*cis*_=10.4 Hz, *J*_3,1*cis*_=1.3 Hz, H_1*cis*_), 5.21 (1H, dd, *J*_2,1*trans*_=17.0 Hz, *J*_3,1*trans*_=1.2 Hz, H_1*trans*_), 5.80 (1H, ddd, *J*_2,1*trans*_=17.0 Hz, *J*_2,1*cis*_=10.4 Hz, *J*_3,2_=5.6 Hz, H_2_); ^13^C-NMR: δ 24.6, 25.2, 25.8, 26.9, 33.7, 51.4, 52.1, 66.9, 73.1, 79.5, 80.6, 81.4, 81.7, 109.1, 112.4, 114.8, 138.5, 156.6; Anal. Calcd for C_18_H_29_NO_7_ (371.43): C 58.21, H 7.87, N 3.77; found C 58.05, H 7.54, N 3.53.

*4,7-Anhydro-2,3-dideoxy-5,6:8,9-di-O-isopropylidene-2(S)-(methoxycarbonylamino)-d-glycero-d-galacto-nononic acid* (**12a**): A solution of **10a** (0.28 g, 0.76 mmol) in methanol (28 mL) was cooled to -78 ^o^C. Ozone was then passed through the solution under vigorous stirring. The maximum time for the ozone treatment was 30 min. This resulted in the formation of a bluish solution. Dry nitrogen was passed through the cold solution in order to remove excess ozone. Ph_3_P (0.20 g, 0.76 mmol) and CH_2_Cl_2_ (11 mL) were added and the solution was allowed to warm up to room temperature while stirring was continued for 1.5 h. The solvent was removed under reduced pressure and the chromatography of the residue (hexane-ethyl acetate, 2:1) afforded 0.25 g (87%) of **11a** as a colorless oil which was used immediately in the next step. ^1^H-NMR: δ 1.32 (3H, s, CH_3_), 1.38 (3H, s, CH_3_), 1.44 (3H, s, CH_3_), 1.47 (3H, s, CH_3_), 2.07-2.17 (1H, m, H_3_), 2.24 (1H, m, H_3_), 3.54 (1H, dd, *J*_8,7_=6.8 Hz, *J*_7,6_=3.7 Hz, H_7_), 3.62-3.68 (1H, m, H_4_), 3.70 (3H, s, CH_3_O), 4.03 (1H, dd, *J*_9,9_=8.7 Hz, *J*_9,8_=4.8 Hz, H_9_), 4.08 (1H, dd, *J*_9,9_=8.7 Hz, *J*_9,8_=6.3 Hz, H_9_), 4.32-4.40 (2H, m, H_2_, H_8_), 4.60 (1H, dd, *J*_6,5_=6.1 Hz, *J*_5,4_=3.7 Hz, H_5_), 4.74 (1H, dd, *J*_6,5_=6.1 Hz, *J*_7,6_=3.7 Hz, H_6_), 5.77 (1H, m, NH), 9.64 (1H, bs, CH=O). A solution of NaClO_2_ (80%, 0.57 g, 6.3 mmol) and NaH_2_PO_4_ (0.71 g, 4.5 mmol) in 3.8 mL of water was added dropwise to a solution of aldehyde **11a** (0.25 g, 0.68 mmol) in acetonitrile/*tert*-butyl alcohol/2-methyl-2-butene (15 mL, 4:4:1) at 0 ^o^C over 5 min and stirred at the same temperature for 35 min. The reaction mixture was poured into brine (12 mL) and extracted with ethyl acetate (2 x 25 mL). The combined organic layers were dried (Na_2_SO_4_). The solvent was removed under reduced pressure and chromatography of the residue on silica gel (hexane-ethyl acetate, 1:2) afforded 0.20 g (74%) of carboxylic acid **12a** as a colorless oil; [α]_d_^25^ = -33 (*c* 0.12, CHCl_3_); ^1^H-NMR: δ 1.31 (3H, s, CH_3_), 1.37 (3H, s, CH_3_), 1.44 (3H, s, CH_3_), 1.45 (1H, s, CH_3_), 2.18-2.26 (2H, m, H_3_), 3.51 (1H, dd, *J*_8,7_=6.8 Hz, *J*_7,6_=3.6 Hz, H_7_), 3.62-3.66 (4H, m, H_4_, CH_3_O), 4.02-4.08 (2H, m, H_9_), 4.37 (1H, ddd, *J*_8,7_=6.8 Hz, *J*_9,8_=6.1 Hz, *J*_9,8_=4.8 Hz, H_8_), 4.48 (1H, m, H_2_), 4.59 (1H, dd, *J*_6,5_=6.1 Hz, *J*_5,4_=3.7 Hz, H_5_), 4.70 (1H, dd, *J*_6,5_=6.1 Hz, *J*_7,6_=3.6 Hz, H_6_), 5.76 (1H, d, *J*_2,NH_=7.9 Hz, NH); ^13^C-NMR: δ 24.6, 25.3, 25.7, 26.9, 30.9, 52.1, 52.3, 66.7, 73.1, 80.0, 2 x 81.0, 81.6, 109.2, 112.5, 156.7, 171.2; Anal. Calcd for C_17_H_27_NO_9_ (389.40): C 52.44, H 6.99, N 3.60; found C 52.10, H 7.00, N 3.41.

*4,7-Anhydro-2,3-dideoxy-5,6:8,9-di-O-isopropylidene-2(R)-(methoxycarbonylamino)-d-glycero-d-galacto-nononic acid* (**12b**): A solution of **10b** (185 mg, 0.498 mmol) in methanol (18 mL) was cooled to -78 ^o^C. Ozone was then passed through the solution under vigorous stirring. The maximum time for the ozone treatment was 30 min. This resulted in the formation of a bluish solution. Dry nitrogen was passed through the cold solution in order to remove excess ozone. Ph_3_P (0.13 g, 0.498 mmol) and CH_2_Cl_2_ (7 mL) were added and the solution was allowed to warm up to room temperature while stirring was continued for 1.5 h. The solvent was removed under reduced pressure and the chromatography of the residue (hexane-ethyl acetate, 2:1) gave 0.16 g (88%) of aldehyde **11b** as a colorless oil which was used immediately in the next step. ^1^H-NMR: δ 1.33 (3H, s, CH_3_), 1.36 (3H, s, CH_3_), 1.43 (3H, s, CH_3_), 1.48 (3H, s, CH_3_), 2.11-2.21 (1H, m, H_3_), 2.26-2.36 (1H, m, H_3_), 3.48 (1H, dd, *J*_8,7_=7.0 Hz, *J*_7,6_=3.6 Hz, H_7_), 3.62-3.67 (1H, m, H_4_), 3.70 (3H, s, CH_3_O), 3.94 (1H, dd, *J*_9,9_=8.7 Hz, *J*_9,8_=4.8 Hz, H_9_), 4.04 (1H, dd, *J*_9,9_=8.7 Hz, *J*_9,8_=6.2 Hz, H_9_), 4.32-4.36 (2H, m, H_2_, H_8_), 4.68 (1H, dd, *J*_6,5_=6.1 Hz, *J*_5,4_= 3.5 Hz, H_5_), 4.74 (1H, dd, *J*_6,5_=6.1 Hz, *J*_7,6_=3.6 Hz, H_6_), 5.63 (1H, m, NH), 9.52 (1H, bs, CH=O). A solution of NaClO_2_ (80%, 0.37 g, 4.1 mmol) and NaH_2_PO_4_ (0.46 g, 2.9 mmol) in 2.5 mL of water was added dropwise to a solution of aldehyde **11b** (0.16 g, 0.43 mmol) in acetonitrile/*tert*-butyl alcohol/2-methyl-2-butene (10 mL, 4:4:1) at 0 ^o^C over 5 min and stirred at the same temperature for 45 min. The reaction mixture was poured into brine (8 mL) and extracted with ethyl acetate (2 x 16 mL). The combined organic layers were dried (Na_2_SO_4_). The solvent was removed under reduced pressure and the chromatography of the residue on silica gel (hexane-ethyl acetate, 1:2) gave 0.12 g (73%) of carboxylic acid **12b** as a white viscous oil; [α]_d_^25^ = -7 (*c* 0.49, CHCl_3_); ^1^H-NMR: δ 1.32 (3H, s, CH_3_), 1.35 (3H, s, CH_3_), 1.42 (3H, s, CH_3_), 1.45 (3H, s, CH_3_), 2.05-2.13 (1H, m, H_3_), 2.29-2.36 (1H, m, H_3_), 3.44 (1H, m, H_7_), 3.60-3.63 (1H, m, H_4_), 3.65 (3H, s, CH_3_O), 3.98-4.04 (2H, m, H_9_), 4.34 (1H, ddd, *J*_8,7_=7.3 Hz, *J*_9,8_=6.2 Hz, *J*_9,8_=5.4 Hz, H_8_), 4.39-4.40 (1H, m, H_2_), 4.70 (2H, m, H_5_, H_6_), 5.61 (1H, d, *J*_2,NH_=7.1 Hz, NH); ^13^C-NMR: δ 24.7, 25.3, 25.8, 26.9, 31.4, 51.9, 52.1, 66.8, 73.1, 80.7, 2 x 81.4, 81.6, 109.0, 112.4, 156.7, 173.4; Anal. Calcd for C_17_H_27_NO_9_ (389.40): C 52.44, H 6.99, N 3.60; found C 52.68, H 7.03, N 3.82.

### Crystal structure determination of **8a**

A single crystal of **8a** suitable for X-ray structure analysis was prepared by growth under slow evaporation of a mixture of diethyl ether and hexane at room temperature in a form of the colorless prisms. The intensities were collected at 295 K on a diffractometer Oxford Diffraction Gemini R CCD using Mo-Kα radiation (0.71073 Å). Details of crystal data, data collection and refinement parameters are given in Table 1.

**Table 1:** Crystal and experimental data for compound **8a**

Empirical formula	C_17_ H_25_ N_1_O_5 _S_1 _
Formula weight	355.46
Temperature, *T* (K)	100 K
Wavelength, λ (Å)	0.71093
Crystal system	Trigonal
Space group	P3121
Unit cell dimensions(Å)	*a* = 10.4026(2) *γ* = 120°
	*b* = 10.4026(2)
	*c* = 30.8227(5)
Unit-cell volume, *V* (Å^3^)	2889(1)
Formula units per unit cell, *Z*	6
Calculated density, *D_x_* (g cm^-3^)	1.233
Absorption coefficient, *μ* (mm^-1^)	0.192
F(000)	744
Crystal size (mm)	0.630 x 0.085 x 0.050
Theta range for data collection, (°)	3.00 - 29.47
Index ranges	-12 ≤ h ≤ 12, -12 ≤ k ≤ 12, -38 ≤ l ≤ 38
Independent reflections [*I*>2σ(*I*)]	3483 (Rint = 0.048)
Absorption correction	Empiric Psi-scan
Max. and min. transmission	0.927 and 0.966
Refinement method	Full-matrix least-squares on F^2^
Data / parameters	3922 / 263
Goodness-of-fit (all)	1.09
Final R indices [*I*>2σ(*I*)]	*R*1 = 0.0480, w*R*2 = 0.0119
R indices (all data)	*R*1 = 0.0527, w*R*2 = 0.1212
Largest diff. peak and hole	0.33 and -0.37 (e Å^-3^)

The structure was solved by direct methods [16]. All non-hydrogen atoms were refined anisotropically by full-matrix least-squares calculations based on F2 [16]. The hydrogen atoms bonded to nitrogen atoms were found in a difference Fourier map and their coordinates and isotropic thermal parameters have been refined freely. All other hydrogen atoms were included in calculated positions as riding atoms, with SHELXL97 [16] defaults. PLATON [17] program was used for structure analysis and molecular and crystal structure drawings preparation. The following crystal structure has been deposited at the Cambridge Crystallographic Data Centre and allocated the deposition number CCDC 697340. These data can be obtained free of charge via www.ccdc.cam.ac.uk/data_request/cif, by emailing data_request@ccdc.cam.ac.uk, or by contacting The Cambridge Crystallographic Data Centre, 12, Union Road, Cambridge CB2 1EZ, UK; fax: +44 1223 336033.
